# Acellular cardiac scaffolds enriched with MSC-derived extracellular vesicles limit ventricular remodelling and exert local and systemic immunomodulation in a myocardial infarction porcine model

**DOI:** 10.7150/thno.72289

**Published:** 2022-06-06

**Authors:** Marta Monguió-Tortajada, Cristina Prat-Vidal, Daina Martínez-Falguera, Albert Teis, Carolina Soler-Botija, Yvan Courageux, Micaela Munizaga-Larroudé, Miriam Moron-Font, Antoni Bayes-Genis, Francesc E. Borràs, Santiago Roura, Carolina Gálvez-Montón

**Affiliations:** 1 ICREC Research Program, Health Science Research Institute Germans Trias i Pujol (IGTP), Can Ruti Campus, Badalona, Spain.; 2 Heart Institute (iCor), Cardiology Department, Germans Trias i Pujol University Hospital, Badalona, Spain.; 3 Cell Therapy Service, Banc de Sang i Teixits (BST), Barcelona, Spain.; 4 Faculty of Medicine, Universitat de Barcelona (UB), Barcelona, Spain.; 5 CIBERCV, Instituto de Salud Carlos III, Madrid, Spain.; 6 Department of Biochemistry, Molecular Biology and Biomedicine, Universitat Autònoma de Barcelona (UAB), Barcelona, Spain.; 7 REMAR-IVECAT Group, Health Science Research Institute Germans Trias i Pujol and Nephrology Service, Germans Trias i Pujol University Hospital, Can Ruti Campus, Badalona, Spain.; 8 Department of Medicine, UAB, Barcelona, Spain.; 9 Department of Cell Biology, Physiology and Immunology, Universitat de Barcelona, Spain.; 10 Faculty of Medicine, University of Vic-Central University of Catalonia (UVic-UCC), Vic, Barcelona 08500, Spain.; 11 Institut d'Investigació Biomèdica de Bellvitge-IDIBELL, L´Hospitalet de Llobregat, Spain.

**Keywords:** extracellular vesicles, cardiac fibrosis, ventricular remodeling, mesenchymal stromal/stem cells, myocardial infarction, swine/pig model, immunomodulation

## Abstract

**Rationale:** Extracellular vesicles (EVs) from mesenchymal stromal cell (MSC) are a potential therapy for cardiac healing after myocardial infarction (MI). Nevertheless, neither their efficient administration nor therapeutic mechanisms are fully elucidated. Here, we evaluate the preclinical efficacy of a tissue engineering approach to locally deliver porcine cardiac adipose tissue MSC-EV (cATMSC-EV) in an acute MI pig model.

**Methods:** After MI by permanent ligation of the coronary artery, pigs (n = 24) were randomized to Untreated or treated groups with a decellularised pericardial scaffold filled with peptide hydrogel and cATMSC-EV purified by size exclusion chromatography (EV-Treated group) or buffer (Control group), placed over the post-infarcted myocardium.

**Results:** After 30 days, cardiac MRI showed an improved cardiac function in EV-Treated animals, with significantly higher right ventricle ejection fraction (+20.8% in EV-Treated; *p* = 0.026), and less ventricle dilatation, indicating less myocardial remodelling. Scar size was reduced, with less fibrosis in the distal myocardium (-42.6% Col I in EV-Treated *vs* Untreated; *p* = 0.03), a 2-fold increase in vascular density (EV-Treated; *p* = 0.019) and less CCL2 transcription in the infarct core. EV-treated animals had less macrophage infiltration in the infarct core (-31.7% of CD163^+^ cells/field in EV-Treated; *p* = 0.026), but 5.8 times more expressing anti-inflammatory CD73 (*p* = 0.015). Systemically, locally delivered cATMSC-EV also triggered a systemic effect, doubling the circulating IL-1ra (*p* = 0.01), and reducing the PBMC rush 2d post-MI, the TNFα and GM-CSF levels at 30d post-MI, and modulating the CD73^+^ and CCR2^+^ monocyte populations, related to immunomodulation and fibrosis modulation.

**Conclusions:** These results highlight the potential of cATMSC-EV in modulating hallmarks of ischemic injury for cardiac repair after MI.

## Introduction

Previous work from our group and others have studied mesenchymal stromal cells (MSC) administration as a therapy for the repair of the injured myocardium after myocardial infarction (MI). In particular, cardiac adipose tissue-derived MSC (cATMSC) are a promising source of therapeutic MSC for MI as acquire *de novo* cardiac and endothelial markers *in vitro* (both human and porcine cells), and contribute to myocardial tissue revascularisation and infarct size reduction *in vivo* 1-4. The initial belief of MSC therapy success was relying on heart tissue replacement by proliferation and differentiation of MSC, but due to the very low, off-site retention and short life of MSC *in vivo*, it has shifted to rather support, such as immunomodulatory, anti-fibrotic and angiogenic functions of MSC to limit MI injury in a paracrine manner 5,6. One of the mechanisms by which MSC are able to promote such pleiotropic effects in distant tissues is the release of extracellular vesicles (EV), transferring molecules to acceptor cells. In fact, accumulating evidence supports the potential of MSC-derived EV (MSC-EV) as a therapy for cardiac healing after MI and also other ischaemia-reperfusion injuries 7-9. In the context of an ischaemic lesion, major issues that arise for therapeutic interventions may include (i) the revascularisation promotion of the affected area, (ii) the management of fibrosis to limit cardiac remodelling and (iii) the modulation of the immune response to switch from an inflammatory to a resolutive post-ischaemic damage. In this context, we previously showed the immunomodulatory, pro-angiogenic and progenitor endothelial cell recruitment capabilities of EV derived from porcine cATMSC *in vitro*, and how tissue engineering enables their high dose, local delivery to the ischemic tissue, where promoted scar vascularisation, reduced macrophage infiltration, and tended to decrease myocardial fibrosis in a 6-day porcine acute MI model 10. Given the promising results of this preliminary study, we now investigated the functional effect of cATMSC-EV delivered within a human decellularised cardiac scaffold in the long-term after acute MI in swine.

## Methods

### EV production and isolation

Primary porcine cATMSC were obtained from pigs (Landrace x Large White) undergoing cardiac surgery (n = 7) by cardiac adipose biopsy (3.3 ± 0.6 g), as previously described 1,10. Cells were cultured in α-MEM medium (Sigma Aldrich) supplemented with 10% heat inactivated foetal bovine serum (FBS), 2 mM L-glutamine, 1% penicillin-streptomycin (all from Gibco Invitrogen Corp.) and 5 μg/mL Plasmocin™ (Invivogen) (α-MEM complete medium).

EV-depleted α-MEM complete medium was obtained by ultracentrifugation of 2x α-MEM complete medium at 100,000×*g* for >16 h (TH641 rotor, adjusted k-Factor = 240.82) in a Sorvall WX Ultra 100 Series ultracentrifuge (Thermo Fisher Scientific). The supernatant was filtered through 0.22-μm (Sarstedt) for sterilisation, and diluted to a 1x working solution with α-MEM medium.

cATMSC-EV batches were harvested from 90 mL of 48-hour supernatant from confluent cATMSC (2.2 × 10^7^ [2.1-2.7 × 10^7^]; ≥ 96% viable, according to trypan blue staining) cultured in EV-depleted α-MEM complete medium, concentrated by ultrafiltration, and purified by size exclusion chromatography (SEC). Specifically, supernatant was sequentially centrifuged at 400×*g* for 5 min and 2000×*g* for 10 min, then concentrated using 15-mL 100-kDa Amicon regenerated cellulose filters (Merck Millipore), and the concentrated cleared supernatant of roughly 1.5 mL was loaded to a SEC column packed with 12 mL of sepharose CL-2B (Sigma-Aldrich). SEC fractions (500 µL each) were eluted and analysed by bead-based flow cytometry to confirm EV (CD63) and MSC (CD44) markers expression. EV-containing fractions were pooled together, obtaining typically 3 mL of purified EV. Also, nanoparticle tracking analysis (NTA) and cryo-electron microscopy were performed to corroborate their nanometric size (211.2 ± 14.7 nm and 109.6 ± 86.3 nm, respectively) and double bilayer, round vesicle structure. All details of EV isolation and characterisation methodology can be found in the previous published work 10 and under the EV200061 repository number.

### EV labelling for *in vivo* tracking

In order to avoid carryover of dye aggregates in the EV preparations, fluorescently-labelled EV were obtained by labelling of the producing cells. cATMSC were stained with the lipophilic NIR815 infrared dye (excitation/emission 786/815 nm; Thermo Fisher Scientific) following manufacturer's instructions, washed twice with PBS (Oxoid) + 2% FBS and then cultured for EV production in EV-depleted medium, as usual. NIR815 EV labelling was routinely corroborated by performing a dot blot assay, placing 5 µL EV/dot (1 µL at a time) in an Amersham Protran 0.45 NC nitrocellulose blotting membrane (GE Healthcare Life Sciences) and reading fluorescence at 800 nm in an Odyssey^®^ CLx Infrared Imaging System (LI-COR Biosciences - GmbH).

### EV delivery within cardiac scaffolds

Cardiac scaffolds were generated by decellularisation of pericardial tissue samples from patients undergoing routine cardiac surgery procedures at our hospital after signed informed consent (n = 14; 12 males, 2 females; mean age 66 ± 18 years; range 51-80 years). The local ethics committee revised and approved this study, whose protocols conformed to the principles outlined in the Declaration of Helsinki. Pericardia were decellularised by a detergent-based protocol (SDS and Triton X-100, Sigma-Aldrich), followed by DNase I (Invitrogen) treatment, lyophilisation, gamma sterilisation (25-35 kGy, Aragogamma, SL) and stored at room temperature (RT) until use, as previously detailed by our group 1,11,12.

The EV pool, corresponding to 2×10^7^ EV-producing cATMSC, was concentrated with a sterile 2-mL 100-kDa Amicon filters (Merck Millipore) to adjust its final volume to 100 µL with 10% sucrose (Sigma Aldrich) as buffer, and then mixed (1:1, v/v) with 0.3% PuraMatrix^®^ self-assembling peptide hydrogel (Corning) in 10% sucrose. In the Control group, 10% sucrose buffer was mixed (1:1, v/v) with the peptide hydrogel instead of cATMSC-EV. Extended methods can be found in **Supplementary Material** and in 10.

### MI pig model

Animal studies were approved by the local Animal Experimentation Unit Ethical Committee and Government Authorities (Generalitat de Catalunya; Code:10078), and comply with guidelines concerning the use of animals in research and teaching as defined by the Guide for the Care and Use of Laboratory Animals. Twenty-four crossbreed Landrace X Large White pigs (32.8 ± 2.0 Kg; 50% female) were pre-anaesthetised with an intramuscular (IM) injection of dexmedetomidine (0.03 mg/kg; Dexdor^®^, Orion Pharma), midazolam (0.3 mg/kg; Laboratorios Normon), and ketamine (0.3 mg/kg; Ketamidor^®^, Richter Pharma AG). Then, anaesthetic induction was performed with an intravenous (IV) bolus of propofol (2 mg/kg; Propovet^®^, Zoetis). Animals underwent endotracheal intubation, and anaesthesia was maintained by 2% isoflurane (IsoVet^®^, BBraun) inhalation. Fentanyl (0.075 mg/kg / 45 min, IV; Fentadon^®^, Dechra) and a 1.5 mg/kg atracurium besylate IV bolus (Sanofi Aventis S.A.) were given during intervention for intra-operative analgesia and muscular relaxation, respectively. After a left lateral thoracotomy in the fourth intercostal space, acute MI was induced by a double-ligation (Optilene 5/0 W-8556 12-S, Ethicon Inc.) of the first marginal branch of the left circumflex artery, 1.5 cm distally from the atrioventricular groove. During MI induction, a continuous IV perfusion of lidocaine (50 µg/kg/min, diluted in 100 mL of NaCl 0.9%; BBraun) was administered to prevent malignant arrhythmias. All animals were monitored under ECG registration to evaluate the ST-segment elevation to confirm the MI induction and evaluate arrhythmic events. After 30 min, animals were blindly randomised to untreated or two different treated groups (**Figure S1**). Treatment consisted on the implantation of the cardiac scaffold rehydrated with hydrogel and containing cATMSC-EV (EV-Treated group; n = 9; EV from 2 × 10^7^ cATMSCs) or buffer (Control group; n = 10), secured over the ischaemic myocardium with surgical glue (Glubran2^®^, Cardiolink S.L.). Additionally, 5 animals were subjected to MI without any scaffold implantation (Untreated group). Tulatromicin (2.5 mg/kg, IM; Draxxin^®^, Pfizer Animal Health) was administered at the end of the surgery as antibiotherapy and a transdermal fentanyl patch was applied to allow analgesic post-operative care (Fentanilo Matrix STADA^®^, STADA). Cardiac troponin I (cTnI) levels were analysed in serum (ARCHITECT STAT High Sensitive Troponin-I; Architect i2000) collected at baseline and 2 h after MI induction to comparatively evaluate myocardial damage between groups (**Figure S1A**).

### Blinding and randomisation

Animals were coded and randomised in blocks (either male or female) to the different study groups using an online random number generator (random.org). Treatment allocation was concealed for all members of the team and animal care staff except for researchers preparing the scaffolds. Only at the time of implantation, 30 min post-MI, the surgeon was informed whether animals were left untreated or a scaffold had to be implanted over the MI scar. Blinding was ensured during the conduct of the experiment and outcome assessment, and groups were finally identified for data analysis.

### Non-invasive cardiac magnetic resonance imaging

Cardiac magnetic resonance imaging (cMRI) was performed at baseline, 2- and 30-days post-MI, before euthanasia (**Figure S1A**). Extended details can be found in **Supplementary Material**.

### Immune response monitoring

Whole blood from pigs was drawn at baseline, 2- and 30-days post-MI in either sodium citrate or serum blood collection tubes (both from BD) for peripheral immune cell or cytokine/chemokine analysis, respectively (**Figure S1A**). On one hand, 100 µL of whole blood were stained with the primary antibodies mouse anti-pig CD14-FITC (BioRad), anti-pig CD16-PE (BioRad), anti-human CCR2 (CD192)-PE-Vio770 (Miltenyi Biotech), rabbit anti-pig CD73 (Novus Biologicals) and mouse anti-pig CD163 (Novus Biologicals). After 15 min incubation at RT, red blood cells were lysed using the Pharm Lyse buffer 10x (BD), washed by centrifugation at 400×*g* for 5 min and resuspended in FACSFlow (BD) with the secondary antibodies donkey anti-rabbit-Cy5 and goat anti-mouse-Cy3 (1:500; Jackson ImmunoResearch). After 15 min incubation at RT, cells were washed with FACSFlow and analysed in an LSR Fortessa flow cytometer (BD). In parallel, 100 µL of whole blood was left unstained, lysed and mixed with Perfect Count Microspheres (Cytognos) to calculate the absolute number of lymphocytes, monocytes and neutrophils according to FSC-A/SSC-A. Details for antibodies and gating strategy used in this study can be found in **Table S1** and **Figure S2**.

On the other hand, serum was frozen at -80 ºC upon collection, and used later on for cytokine/chemokine analysis. Succinctly, serum was thawed in ice, centrifuged for debris pelleting, and the levels of granulocyte-macrophage colony-stimulating factor (GM-CSF), IFNγ, IL-1α, IL-1β, IL-1ra, IL-2, IL-4, IL-6, IL-8, IL-10, IL-12, IL-18 and TNF-α were analysed using the Milliplex MAP Porcine Cytokine/Chemokine Magnetic Bead Kit (PCYTMG-23K-13PX; Millipore) following the manufacturer's instructions in a Luminex 200 instrument (Luminex Corporation).

### Tissue collection and morphometric analysis

All animals were euthanised through a pentobarbital sodium overdose (200 mg/kg, IV; Dolethal^®^, Vetoquinol E.V.S.A) at 30 days of follow-up. After mid sternotomy, excised hearts were washed in saline buffered solution to remove any residual blood, then scanned at 700 and 800 nm in a Pearl Impulse Imager (LI-COR), sliced transversely into three 1-1.5 cm sections (S1-S3) from artery ligation to the apex, scanned again at 700 and 800 nm for cATMSC-EV tracking within heart sections, and digitally photographed for morphometric analysis.

Then, tissue transverse samples (~5 mm) from the middle of the scar (infarct core) and from the septum non-infarcted wall (distal myocardium) of each section were obtained, fixed in 10% buffered formalin and embedded in paraffin; snap-frozen in OCT; or snap-frozen in N_2_(L) for histopathological, immunohistofluorescence and transcriptomic evaluation, respectively.

### Histopathological analysis

Masson's trichrome and modified Movat's pentachrome stainings were done on 4 µm-thick paraffin sections for the primary histological examination and scar fibrosis evaluation. Picrosirius Red staining was performed to analyse interstitial collagen deposition in the MI scar and remote myocardium. Collagen type I and III presence was analysed in 4 random fields of each tissue section imaging with polarized light in a DMI6000B microscopy (Leica), and quantified using Image-Pro Plus software (6.2.1 version; Media Cybernetics, Inc.).

### Immunohistofluorescence analysis

Vascular density was blindly evaluated in the infarct core, distal area and scaffolds of 10-μm OCT-embedded tissue cryosections immunolabelled with biotinylated *Griffonia simplicifolia* Lectin I isolectinB4 (IsoB4; 1:25; Vector Labs) and Streptavidin-Alexa Fluor 488 (1:500; Invitrogen). Representative images were taken in slides labelled together with rabbit anti-pig elastin (1:100; Abcam), goat anti-human cTnI (1:100; Abcam), and 4′,6-diamidino-2-phenylindole dihydrochloride (DAPI; 1:1000; Sigma-Aldrich) to counterstain the nuclei.

Tissue immune infiltration was studied by staining for porcine CD3, CD25 (1:100; AbD Serotec), CD163, CD73 (1:100; Novus Biologicals), and cTnI to counterstain the myocardium. Cy2, Cy3 (1:1000; Jackson ImmunoResearch Laboratories), or Alexa Fluor 488 and 647 (1:1000; Molecular Probes) were used as secondary antibodies and nuclei were counterstained with DAPI. Details for antibodies used in this study can be found in **Table S1**.

All images were acquired in an Axio-Observer Z1 confocal microscope (Zeiss). Quantitative measurements of blood vessel area (IsoB4 staining) in either the infarct core or scaffold were performed using Image-Pro Plus software in 4 different random fields per section. Quantification of CD163^+^, CD73^+^, CD3^+^ and CD25^+^ cells was carried out by two independent investigators measuring at least 4 different optical fields from each section (S1-S3).

### Transcriptomic evaluation

Snap-frozen tissue from the infarct core (71.9 ± 15.2 mg) or distal area of the myocardium (74.2 ± 15.9 mg) from sections S1 or S2 were homogenised in cold TriPure Isolation reagent (Merck) using the TissueRuptor (Qiagen). Then, the RNA, DNA and protein fractions were isolated following the TriPure manufacturer's protocol. Both RNA concentration and quality were evaluated with a NanoDrop 1000 spectrophotometer (Thermo Fisher Scientific), and 10 µg were retrotranscribed to cDNA using the High Capacity cDNA Reverse Transcription kit (Applied Biosystems). Then, the expression levels of IL-10, TNF-α, CCL2, TGF-β1, TGF-β3, LRP1, MMP2, MMP9, TIMP1, GAPDH, PGK1 and GUSB were assessed using the corresponding porcine TaqMan^®^ FAM-MGB probes (**Table S2**) and the TaqMan^®^ Fast Advances Master Mix (Thermo Fisher Scientific) in a LightCycler^®^ 480 Real-time PCR system (Roche). The expression of all genes was analysed using the 2^-ΔCt^ method against the 3 candidate endogenous genes evaluated (GAPDH, PGK1, and GUSB). PGK1 was chosen as the most suitable for normalisation according to the differential expression in the infarct core compared to the distal tissues in the Untreated group. Then, the 2^-ΔΔCt^ expression level was calculated for each gene normalized to PGK1, and compared to the expression of the corresponding section (S1 or S2) of the Untreated group.

### Functional enrichment analysis of decellularised scaffolds

We performed a functional enrichment analysis of the proteomic dataset we previously published 13 focusing on the proteomic content of decellularised pericardial scaffolds using the PANTHER software (version 17.0 Released 2022-02-22). We performed an Overrepresentation Test annotating for “PANTHER GO-slim Biological Process” and “GO molecular function complete” against *Homo sapiens* (all genes in database) as a reference list, using the Fisher's exact test with FDR correction. Then, we manually curated the dataset looking for cardiac fibrosis and tissue healing-related proteins based on literature search.

### Statistical analysis

Data is shown as mean with SD unless stated otherwise. Statistical differences were considered significant when *p* < 0.05 applying the appropriate statistical tests for each dataset after checking for normality of data, and indicated in each figure legend. Analysis was performed using Graphpad Prism (9.0.1 version) and SPSS 21.0.0.0 (SPSS, Inc.) softwares.

Statistical significances in normalised data such as gene expression in ΔΔCt was calculated using a One-sample T-test if passed the Kolmogorov-Smirnoff normality test or the Wilcoxon Signed Rank Test for nonparametric data, to the theoretical mean of “1.0” of the Untreated group. Differences between the Control and EV-Treated groups were calculated with a Student T-test.

## Results

### Animal experimentation

A total of 24 pigs were used in this study. Four animals (n = 1 Untreated; n = 2 Control; n = 1 EV-Treated groups) died during MI induction or 48 h post-MI cMRI scan due to ventricular fibrillation. Two animals were lacking full cMRI (n = 1 Control; n = 1 EV-Treated), one of which (n = 1 Control) was also lacking its corresponding necropsy as it could not be performed due to COVID19 lockdown at our institution. Therefore, final data included 4 animals in the Untreated group, 7 animals in the Control group, and 8 animals (n = 7 including cMRI) in the EV-Treated group (see **Figure S1B**). In terms of safety, no animal showed local inflammatory reaction, infection signs nor graft-versus-host disease. In all animals with scaffold implantation, the construct was seen covering the infarcted area after 30 days of follow up. Serum cTnI analysis post-MI indicated similar myocardial damage in all groups (*p* = 0.153 paired two-way ANOVA; **Table S3**).

### Cardiac function analysis

**Figure 1:**
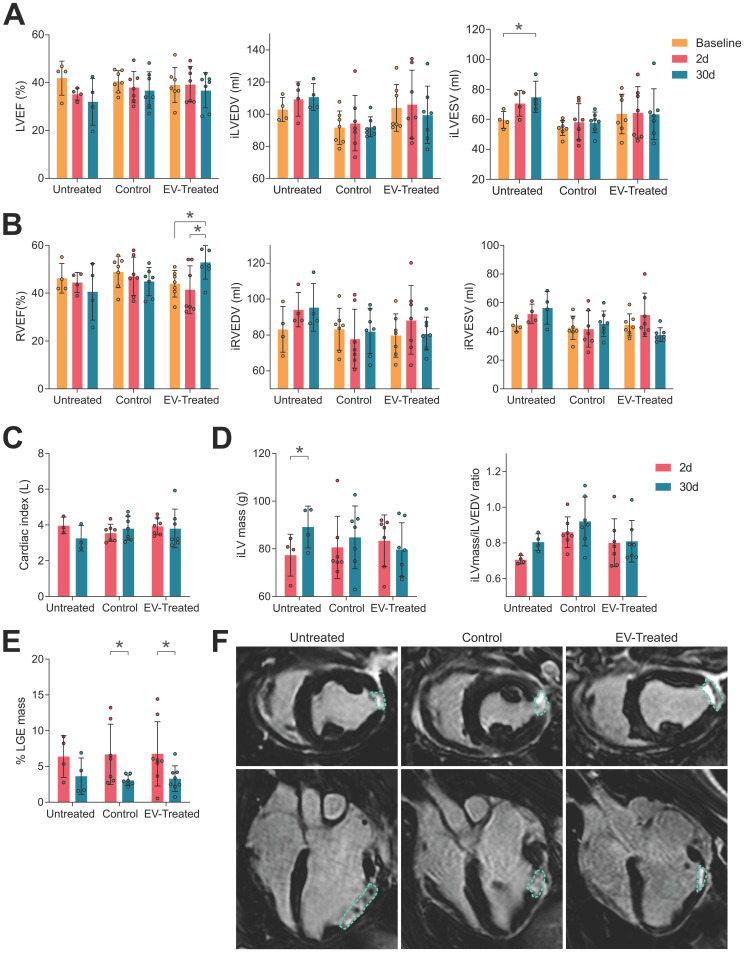
**cMRI analysis indicated an improvement in cardiac function by scaffold implantation.** (**A**) The left ventricular ejection fraction (LVEF, left), indexed left ventricular end-diastolic and systolic volumes (iLVEDV, middle; iLVESV, right) were maintained by scaffold implantation. (**B**) Right ventricular ejection fraction (RVEF, right) was significantly improved by cATMSC-EV administration; with the indexed right ventricular end-diastolic and systolic volumes (iRVEDV, middle; iRVESV, right) maintained. (**C**) The cardiac index of animals was maintained by scaffold implantation. (**D**) LV hypertrophy was avoided in treated animals according to the iLV mass (left) to the end-diastolic volume (EDV) ratio (right). (**E**) The infarct size was reduced after 30 days of follow up in treated animals according to the percentage of LGE mass. Bars indicate mean ± SD and each data point corresponds to an animal of each group (n = 4;7;7, respectively). Statistical differences were calculated using a paired Two-way ANOVA with Tukey's posthoc analysis. (**F**) Representative LGE cMRI images in short (upper pannel) and long (bottom) axis views at the area of the infarct scar at 30 days post-MI. The infarct area appears in white (circled), while the non-infarcted myocardium appears black.

Baseline cMRI data showed no differences in cardiac function between the studied groups (**Table S4**) nor in infarct size (% LGE mass of 6.4 ± 2.9 in Untreated, 6.7 ± 4.2 in Control and 6.8 ± 4.5 in EV-Treated animals). Thirty days post-MI, pigs with implanted tissue engineering constructs were those with better cardiac function. Untreated animals displayed a decrease in LVEF and RVEF, not significant due to the small animal number, and a tendency towards dilatation indicated by the increase in end-diastolic and -systolic volumes of the left (iLVEDV and iLVESV) and right ventricle (iRVEDV and iRVESV) (**Figure 1A and 1B**). Scaffold implantation managed to conserve iLVEDV and iLVESV, resulting to a preserved LVEF in both Control and EV-Treated groups (-19,0% in Untreated; -6,6% in Control and -4,2% in EV-Treated animals of LVEF at 30 days post-MI compared to Baseline; **Table S4**). Preservation of LVEF translated to a conserved cardiac index (**Figure 1C** and **Table S4**). Moreover, EV-Treated pigs were able to significantly improve the RVEF (-9,4% in Untreated; -6,7% in Control and +20,8% in EV-Treated animals of RVEF at 30 days post-MI compared to Baseline; **Table S4**) by maintaining iRVEDV and iRVESV (**Figure 1B**).

In terms of maladaptive remodelling, cATMSC-EV treatment reduced LV hypertrophy and LV myocardial remodelling as they managed to maintain the ratio of LV mass to LV end-diastolic volume (**Figure 1C**) and avoided the increase in the EDV and ESV of both ventricles (**Figure 1A, B**). Finally, a significant reduction in scar size was observed in the EV-Treated (6.8 ± 4.5% LGE mass at 2 days* vs* 3.3 ± 1.8 at 30 days post-MI; *p* = 0.028) and Control (6.7 ± 4.2% *vs* 3.1 ± 0.7%; *p* = 0.032) groups according to the percentage of LGE mass compared to untreated pigs (6.4 ± 2.9% *vs* 3.6 ± 2.5%; *p* = 0.32) (**Figure 1E-F**).

### cATMSC-EV *in vivo* tracking

**Figure 2:**
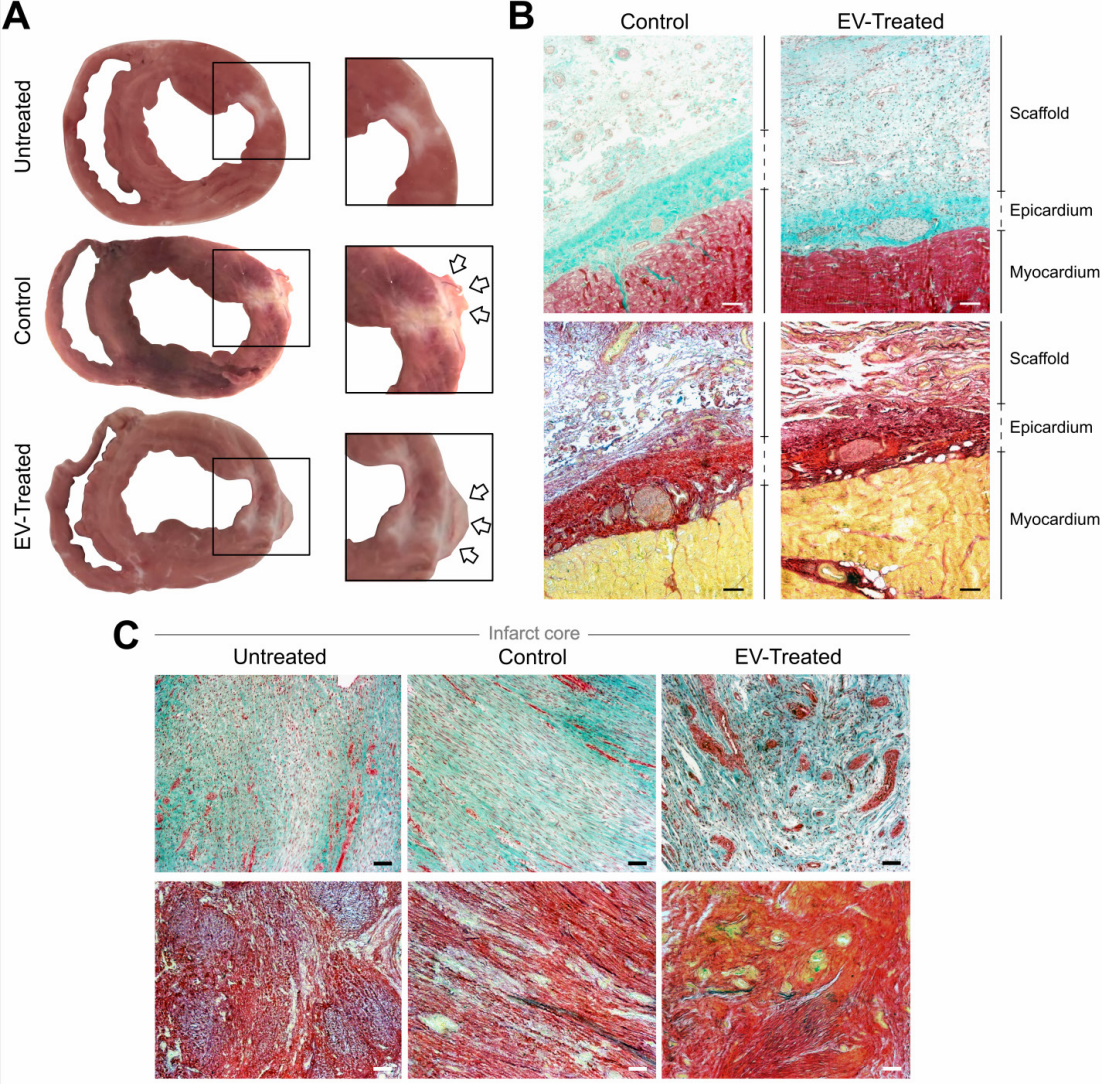
** Morphological and histological analysis.** (**A**) Representative sections of Untreated, Control and EV-Treated pigs at 30 days post-MI and their respective enlarged images (right) showing the implanted scaffolds (arrows) covering the ischemic scar. (**B, C**) Masson's trichrome (top) and modified Movat's pentachrome (bottom) staining microphotographs of (**B**) representative Control and EV-Treated animals, showing the correct adhesion of the cardiac constructs over the epicardium and (**C**) the infarct core of the 3 groups of the study. Scale bars = 50 µm.

Animals were euthanized after 30.2 ± 1.2 days and hearts were examined, showing that the scaffolds implanted in all Control and EV-Treated pigs adhered and integrated to the underlying myocardium, and covered the ischaemic scar (**Figure 2A-B**). Whole hearts and heart sections were scanned at 700 and 800 nm to track NIR815-labelled cATMSC-EV, but no signal attributable to NIR815 dye could be detected at 30 days post-MI (data not shown), while it was previously detected after 6 days 10. There were no differences between labelled, unlabelled and control scaffolds fluorescent signal, indicating the overlapping 700 and 800 nm signal was due to tissue autofluorescence, much higher in the scaffolds and fibrotic scar.

### Myocardial vessel density and scar fibrosis

**Figure 3:**
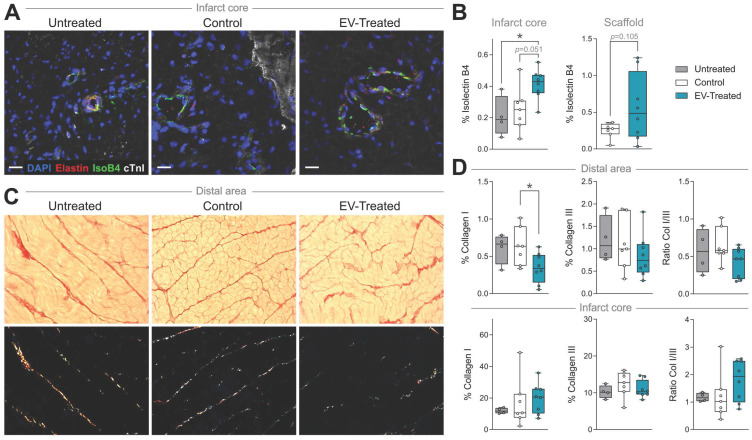
** Vascularisation is improved and distal fibrosis is reduced in EV-Treated animals.** (**A**) Representative immunohistofluorescence images of IsolectinB4 (IsoB4; green), elastin (red), cardiac troponin I (cTnI; white) and DAPI (blue) in the infarct core at 30 days post-MI. (**B**) Percentage of vessel area within the infarct core (left) and scaffold (right). (**C**) Representative microphotographs of Picrosirius Red staining distinguishing the collagen (red) and cardiac muscle (yellow) under bright field (upper panels), and polarized light (bottom) exhibiting collagen I (red/yellow) and collagen III (green) fibrils in the same sections of distal myocardium of each experimental group. (**D**) Percentage of collagen I, III and col I/III ratio in distal (upper panel) and infarct (bottom) areas of the 3 groups of the study. Tukey boxplots from n = 4;7;8 animals in Untreated, Control and EV-

In particular, cATMSC-EV implantation significantly increased the vascularised area in the infarct core of EV-Treated animals compared to Untreated and Control groups (0.41 ± 0.10 in EV-Treated animals compared to 0.21 ± 0.13 in Untreated; *p* = 0.034, and 0.25 ± 0.14 in Control; *p* = 0.051) (**Figure 2C, 3A-B**), and there was a tendency on promoting vascularisation within the implanted scaffolds filled with cATMSC-EV relative to control scaffolds (0.56 ± 0.45 in EV-Treated and 0.25 ± 0.10 in Control; *p* = 0.105). In distal areas, no differences between groups were found (data not shown).

**Figure 4:**
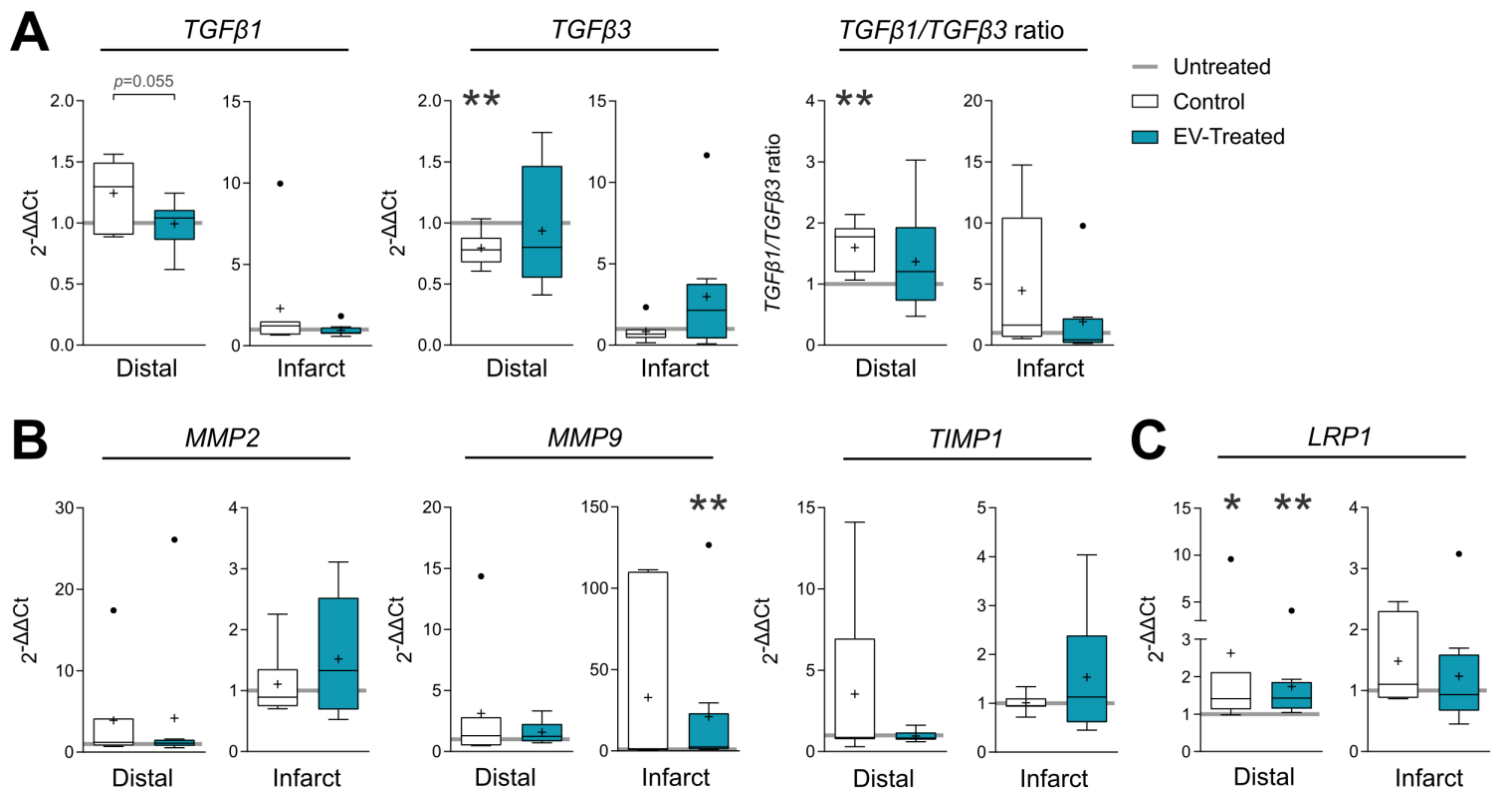
** Gene expression of fibrosis drivers and remodelling-related genes in the distal tissue or infarct core 30 days post-MI.** Gene expression was calculated using the ΔΔCt method relative to the endogenous gene PGK1 and to the expression level of the untreated animals. Tukey boxplots with the mean shown as “+” and outliers as a point, from n = 4;7;8 animals in Untreated, Control and EV-Treated groups, respectively. Statistical significance to the Untreated group was calculated using a One-sample T-test and indicated as *p < 0.05; **p < 0.01. Differences between the Control and EV-Treated groups were calculated with a Student T-test.

In terms of fibrosis, Picrosirius Red staining revealed a reduced collagen I deposition in distal myocardium of EV-Treated animals at 30 days post-MI (**Figure 3C-D**). Also, there was a tendency towards a decrease in collagen III and collagen I/III ratio, collectively indicating a decreased fibrosis in distal myocardium of EV-Treated animals. In the infarct core, a mild increase in collagen I and I/III ratio were observed in EV-Treated animals, although there were no statistical differences detected between groups. We next studied known drivers and modulators of fibrosis, such as the TGF-β pathways and metallopeptidases (MMP) activity. There was an increased expression of TGF-β1 and decrease of TGF-β3 in the distal area of Control animals (**Figure 4A**), with a mild increased transcription of the fibrosis regulators MMP2, MMP9 and TIMP1 (**Figure 4B**), while the addition of cATMSC-EV reverted this scaffold-associated effect. Only LRP1 was significantly increased in the distal area of both Control and EV-Treated animals (**Figure 4C**). In the infarct core, MMP2, MMP9 and TIMP1 expression levels were higher in EV-Treated compared to Untreated animals (**Figure 4B**).

### Inflammatory response

**Figure 5:**
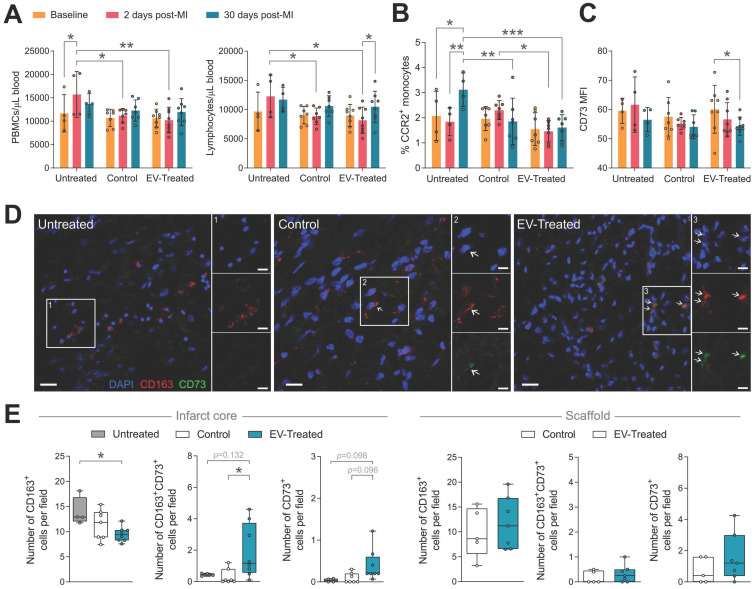
** Scaffold implantation modulates the systemic immune response post-MI, and cATMSC-EV promote a local effect.** (**A-C**) Systemic analysis of the immune response post-MI. (**A**) The increase in the number of total peripheral blood mononuclear cells (PBMC) (left) and specifically in the number of lymphocytes (right) in whole blood that occurs 2 days after MI (red) is prevented by scaffold implantation. (**B**) The increase in the number of CCR2^+^ monocytes observed 30 days post-MI in untreated animals is blocked by scaffold implantation. (**C**) EV-Treated animals reduce the expression of CD73 in circulating monocytes 30 days from MI. Bars indicate mean ± SD and each data point corresponds to one animal (n = 4;7;8 respectively). Statistical significance was calculated with a paired Two-way ANOVA with Tukey's posthoc analysis. (**D-E**) Representative images (**D**) and quantification (**E**) of the number of infiltrating macrophages (CD163^+^; left), of which expressed CD73 (middle), and cells expressing CD73 (right) by immunohistofluorescence analysis at 30 days post-MI. Statistical significance was calculated with a One-way ANOVA with Tukey's posthoc analysis (Infarct core, left) and Student T-test (Scaffold, right). (**E**) CD163 (red), CD73 (green) and DAPI (blue).

In relation to the modulation of the post-MI inflammatory response, the local implantation of the scaffold -regardless of cATMSC-EV co-delivery-, triggered a systemic effect, preventing the peripheral blood mononuclear cells (PBMC) rush happening 2-days post-MI, mainly constituted by lymphocytes (**Figure 5A**). Of note, no changes were observed in neutrophil counts (data not shown). Scaffold implantation also regulated circulating monocyte phenotype. Treatment blocked the surge of circulating CCR2^+^ activated monocytes at 30 days post-MI (**Figure 5B**), and cATMSC-EV delivery significantly reduced CD73 expression on peripheral monocytes (**Figure 5C**). Moreover, the number of infiltrating macrophages (CD163^+^) in the infarct core was significantly less in EV-Treated animals (**Figure 5D-E**), but more of these fewer macrophages went on to express CD73. Also, some CD73^+^ cells appeared in the infarct core and scaffold of EV-Treated animals. Of note, we did not observe differences in the amount of infiltrating CD3^+^ nor CD25^+^ lymphocytes (data not shown).

**Figure 6:**
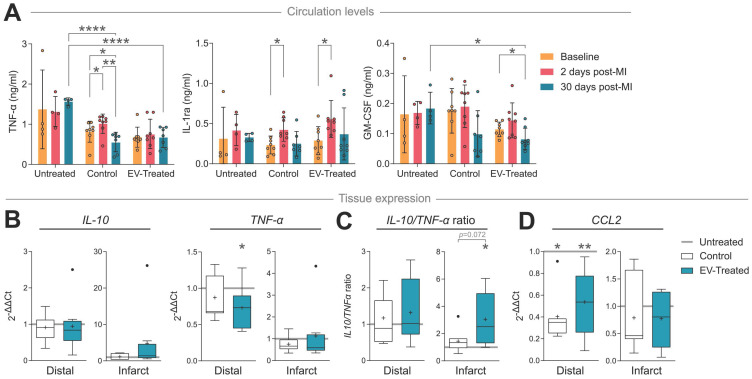
** cATMSC-EV delivery induces an anti-inflammatory cytokine profile and reduces macrophage recruitment chemokine expression in circulation and in the infarct core or distal tissue 30 days post-MI.** (**A**) TNF-α, IL-1ra and GM-CSF serum levels at baseline, 2 days or 30 days post-MI. Statistical significance indicated according to Two-way ANOVA with Tukey's post-hoc test. (**B-D**) Gene expression of cytokines IL-10, TNF-α (**B, C**) and chemokine CCL2/MCP-1 (**D**) calculated using the ΔΔCt method relative to the endogenous gene PGK1 and to the expression level of the Untreated group. Tukey boxplots with the mean shown as “+” and outliers as a point, from n = 4;7;8 animals in Untreated, Control and EV-Treated groups, respectively.

In terms of cytokine/chemokine response, TNF-α circulation levels increased 2 days after scaffold implantation in Control animals (*p* = 0.014), which was avoided by cATMSC-EV addition (**Figure 6A**), and normalised after 30 days of follow up (*p* = 0.003). Notably, TNF-α levels in Control and EV-Treated animals were significantly lower compared to Untreated animals at 30 days post-MI (0.56 ± 0.24 ng/mL in Control and 0.67 ± 0.25 ng/mL in EV-Treated animals compared to 1.57 ± 0.09 ng/mL in Untreated; *p* < 0.0001). Scaffold implantation -regardless of cATMSC-EV co-delivery-, also managed to increase circulating IL-1 receptor antagonist (IL-1ra) levels 2 days post-MI, but only EV-Treated animals showed significant reduction in the amount of GM-CSF present in serum 30 days post-MI. Of note, there were no relevant changes in the expression of other cytokines analysed (IFNγ, IL-1α, IL-1β, IL-2, IL-4, IL-6, IL-8, IL-10, IL-12 and IL-18; data not shown).

Locally, cATMSC-EV implantation fostered IL-10 transcription in the infarct core and reduced TNF-α in the distal tissue (**Figure 6B**), which induced an anti-inflammatory profile according to an increased IL-10/ TNF-α ratio in distal but especially infarcted tissue, not seen in Control animals (**Figure 6C**). Also, the expression of the CCR2^+^ macrophage recruitment chemokine CCL2/MCP-1 was markedly reduced in Control and EV-Treated animals, both in infarct core and distal tissue (**Figure 6D**).

### Functional enrichment analysis of decellularised scaffolds

Given the beneficial effects observed in both the Control and EV-Treated groups we decided to study the molecular composition of decellularised scaffolds. Gene ontology was used to identify the molecular functions and biological pathways enriched in decellularised human pericardial scaffolds that could be mediating the *in vivo* effects after its implantation. Based on the previously published dataset 13, we could identify the expected ubiquitous presence of extracellular matrix (ECM) proteins as previously reported, with structural and binding activity as main molecular functions (**Figure S3A**), such as to growth factors, glycosaminoglycans or integrins for cell adhesion. As pointed out previously 13, decellularised pericardial scaffolds bear fibronectin, collagens (I-VI, XI, XII, XIV, XXI) and heparan sulphate proteoglycans, amongst others, that can mediate cell survival and adhesion, and in this case, also EV retention for efficient administration. In terms of biological processes (**Figure S3B**), we could detect enriched proteins related to wound healing, tissue regeneration and response to wounding. Then, a close inspection of the dataset revealed the presence of (i) the hyalectan versican, able to bind ECM components, chemokines, growth factors, CD44 (present in cATMSC-EV), and capable to modulate a wide range of cellular responses, such as cell inflammatory activation; (ii) the antioxidant agent thioredoxin, a potent cardioprotective against ischemia-reperfusion injury 14,15; and (iii) the small leucine-rich proteoglycans class I biglycan, decorin and class II lumican, crucial to reduce cardiac fibrosis and dysfunction 16.

### Sex differences

After subgroup analysis based on the sex of the animals, some differences between males and females were detected. In terms of cardiac function, male sex was a beneficial factor in EV-Treated animals for the observed improvement in RVEF (relative increase of +49%, from 33.6 ± 1.0 at 2 days to 50.1 ± 7.4 at 30 days, *p* = 0.006 in male pigs *vs* +9% from 51.8 ± 3.9 at 2 days to 56.6 ± 5.1 at 30 days, *p* = 0.663 in female pigs), while there was no sex-dependent effect in infarct scar size reduction by EV administration (relative decrease of -52%, from 8.2 ± 6.4 at 2 days to 4.0 ± 2.5 at 30 days, *p* = 0.148 in male pigs *vs* -51% from 5.4 ± 1.1 at 2 days to 2.6 ± 0.5 at 30 days, *p* = 0.573 in female pigs). On the other hand, we observed 35% more reduction of % LGE mass in male animals compared to female animals upon scaffold implantation in the Control group (relative decrease of -61%, from 7.5 ± 4.8 at 2 days to 2.9 ± 0.7 at 30 days, *p* = 0.056 in male pigs *vs* -27% from 4.8 ± 2.5 at 2 days to 3.5 ± 0.8 at 30 days, *p* = 0.996 in female pigs).

Additionally, regarding the post-MI inflammatory response, TNF-α at 30 days post-MI was specifically induced in Untreated male animals and its expression reduced by EV administration. At the same time, EV-induced IL-1ra secretion at 2 days post-MI was especially promoted in male animals.

## Discussion

In this study, we evaluated the long-term benefits of cATMSC-EV delivery in a porcine model of MI using a tissue engineering construct in terms of cardiac function and tissular, cellular and molecular responses. We showed the therapeutic *in vivo* effect on LVEDV, LVEF and cardiac index preservation, improvement of RVEF and reduction of myocardial scar size and cardiac remodelling avoiding LV hypertrophy. Remarkably, cATMSC-EV administration in a cardiac scaffold also modulated both systemically and locally the expression of inflammatory mediators and fibrosis modulators.

In contrast to individual secreted factors, EV deliver a targeted, protected package of diverse, active biomolecules that can target various pathways at once, necessary for complex scenarios such as the post-infarcted myocardium. Consequently, the mechanism of action of EV is multifactorial and not limited to a singular signalling pathway, functional or host cell type. In this context, we previously described the immunomodulatory, pro-angiogenic and progenitor endothelial cell recruitment capabilities of porcine cATMSC-EV *in vitro* and how they promoted scar vascularisation and reduced fibrosis, macrophage and lymphocyte infiltration in a short-spanned porcine model of acute MI 10. In the present long-term study, a significant reduction in scar size was observed in EV-Treated animals, with less inflammatory profile and increased vascular density. The non-infarcted myocardium of Treated animals displayed also less inflammatory profile, and more elastic characteristics.

While the immunomodulatory functions of cATMSC-EV were previously confirmed *in vitro* 10, the effects that were observed here *in vivo* involve different cells and targets that shed light into their function mechanisms. On one hand, we found correlation between the *in vitro* immunomodulation of the T cell response by MSC-EV 17 with the reduction in T cell activation 6 days post-MI 10*,* but no changes were seen on T cell infiltrate nor activation according to CD25 staining at 30 days. On the other hand, while monocyte phenotype was not modified *in vitro* by MSC-EV 17,18, we found a marked impact on macrophage populations *in vivo* at 30 days post-MI. The fact that EV interact with the whole organism instead of isolated PBMC and that they are combined with the acellular cardiac scaffold could explain these differences. More specifically, we observed a pronounced alteration in monocyte/macrophage dynamics, an important cell target for tissue repair after MI, as they are responsible for the switch from inflammatory to reparative phases, and involved in fibrosis establishment 19,20. cATMSC-EV implantation managed to reduce the amount of CCR2^+^ inflammatory macrophages and their tissue infiltration, as we already observed 6 days post-MI 10. The mechanism behind this effect may be the reduction in the circulating levels of monocyte chemoattractant and stimulating factor GM-CSF 21,21 and of CCL2/MCP-1 tissue expression, which triggers inflammatory CCR2^+^ monocyte recruitment to the heart 22. The CCL2-CCR2 axis has been described to be a key factor in resolution of ischaemic injury 23, and involved in ischaemic cardiac fibrosis 24,25. For instance, CCL2 may enhance the fibrogenic potential of macrophages by inducing TGF-β1 and collagen synthesis 26, is able to also recruit fibroblasts progenitors and to modulate their MMP expression 24, and the abundance of CCR2^+^ macrophage is associated with LV remodeling and systolic function in heart failure patients 27.

On the other side, the increase in infiltrating CD73^+^ macrophages correlated to a lower number of circulating CD73^+^ monocytes in EV-Treated animals, suggesting a recruitment of this anti-inflammatory cell subset to the myocardium. More specifically, CD73 is responsible for the hydrolysis of ADP/AMP to adenosine, reducing the pool of pro-inflammatory ATP, present extracellularly post-ischaemia and increasing the amounts of anti-inflammatory adenosine 28,29. In turn, CD73 is also implicated in vascularisation promotion, which might explain the increase in vascular density found in myocardial scar of EV-Treated animals 30. Also, CD73 presence was found crucial for cardiac healing after MI, previously described to profoundly alter T cell response 31 and key for MSC-mediated MI repair after implantation with a hydrogel support 32. Particularly, macrophage CD73 expression seems to be induced in a longer time point, indicative of the reparative phase, as we already found an upregulation of CD73 expression in scar-infiltrating macrophages 30 days post-MI after MSC administration 33, but was not yet present 6 days post-MI after cATMSC-EV therapy 10.

Molecularly, cATMSC-EV implantation in the engineered scaffold regulated the inflammatory cytokine production that occurs post-MI, promoting the decrease in inflammatory TNF-α, the increase in IL-1ra, a natural competitive antagonist of the IL-1 receptor, blocking IL-1α/β inflammatory effect 34 and induced a local increased expression of anti-inflammatory IL-10. The reduction of inflammatory mediators can then modulate both expression and activity of TGF-β, metallopeptidases and other profibrotic mediators, to reduce collagen synthesis and deposition. EV treatment seems to have speed up the MI repair process, switching faster from the inflammatory phase to the reparative phase than Untreated animals.

The significant changes in the inflammatory processes promoted by cATMSC-EV delivered in cardiac scaffolds could explain the marked decrease in infarct size from 2 days to 30 days, as immunomodulation by MSC-EV is a common mechanism towards tissue regeneration 35. Infarct size defined by LGE mass would quantify not only the ischaemic tissue but also the acute oedema present at 2 days post-MI, which significantly decreased 30 days after scaffold implantation. This may indicate that cATMSC-EV delivered in acellular cardiac scaffolds manage to reduce the inflammatory-induced tissue damage to keep the affected area limited to the ischaemic injury. Moreover, the reduction of collagen I in the myocardial scar previously observed in EV-Treated animals after 6 days of follow up 10 could be also a trigger for long-term infarct scar size reduction. We acknowledge the limitation in the low number of Untreated animals (n = 4) that leads to a lack of statistical differences in cardiac function in the MI model, which can weaken the power of our observations. Nevertheless, the implanted tissue engineering constructs finally reduced the overall size of MI, prompting to a positive benefit in global cardiac remodelling, as demonstrated by different parameters such as a trend to avoid increase in ventricle dilatation, maintenance of LVEF and cardiac index, improvement of RVEF and a reduction in LV hypertrophy. Whether those effects are simply explained by the changes detected locally at the region of treatment or whether are also explained by the improvement in systemic inflammation after treatment needs to be further explored. Remarkably, in this regard, we observed that local cATMSC-EV delivery exerts a notable effect on global cardiac remodelling avoiding the fibrotic deposition, and improving the overall function of the remote myocardium, as expressed by the increase in RVEF.

EV efficient administration is a matter of debate as systemic injection leads to poor retention in the target tissue, needing repeated injections of high EV doses to obtain significant benefit 36 and big production costs if we take MSCs doses used previously in clinical trials as reference 37,38. Also, their efficacy can be distinct depending on the route of administration 39-41. In this sense, we show how local administration using cardiac scaffolds can lower the number of EV needed for an active dose, observing a beneficial effect already with 20 × 10^6^ MSC-EV-producing cells/animal, that also modulated the systemic immune response as when administered systemically. We could detect the signal delivered by the encapsulated cATMSC-EV specifically in the infarcted tissue after 6 days from MI, most probably corresponding to dye being incorporated to the cells capturing the labelled EV, and indistinguishable at 30 days. We speculate that the EV effect is in fact a trigger for immunomodulation and activator of the endogenous repair mechanisms, to which the scaffold helps by constituting a niche for pro-regenerative cell grafting 10. Indeed, we noticed a positive functional improvement after EV-free scaffold implantation as observed before 42 and a reduced circulatory leukocytes and inflammatory cytokine response, which highlights the power of ECM components. The ECM not only offers structural integrity for cardiac repair, but also harvests bioactive components known to influence the bioavailability of growth factors and cytokines, autophagy, angiogenesis, inflammation and cell migration 16,43. Amongst them, we were able to identify adhesion molecules for cells and EV and specific mediators of cardioprotection and modulators of cardiac fibrosis in the decellularised pericardial scaffolds^13^, which can mediate in part the *in vivo* effects observed after scaffold implantation. At the same time, the addition of cATMSC-EV can confer the added cell recruitment, pro-angiogenic and immunomodulation properties to the scaffold for enhanced therapy. This cardiac scaffold was already a successful combination with cATMSC at pre-clinical level 1,2,13,44. To note, these studies found a reduction in infarct size and improvement in cardiac function in animals treated with scaffolds filled with cATMSC that are comparable to the present results obtained with cATMSC-EV administration.

## Conclusions

In summary, our data demonstrate the potential of multifunctional cATMSC-EV combined with acellular cardiac scaffolds over post-infarcted myocardial tissue. As we reported previously, MSC-EV showed an anti-inflammatory profile both at local and systemic levels, as well as promote myocardial vascularisation and fibrosis reduction, and avoid global cardiac adverse remodelling *in vivo*. Taken together, these results highlight the immunomodulatory potential of cATMSC-EV in the long-term progress of ischaemic injury and reduction of infarct scar size for cardiac healing after MI.

## Supplementary Material

Supplementary figures, tables, and methods.

## Figures and Tables

**Figure 7:**
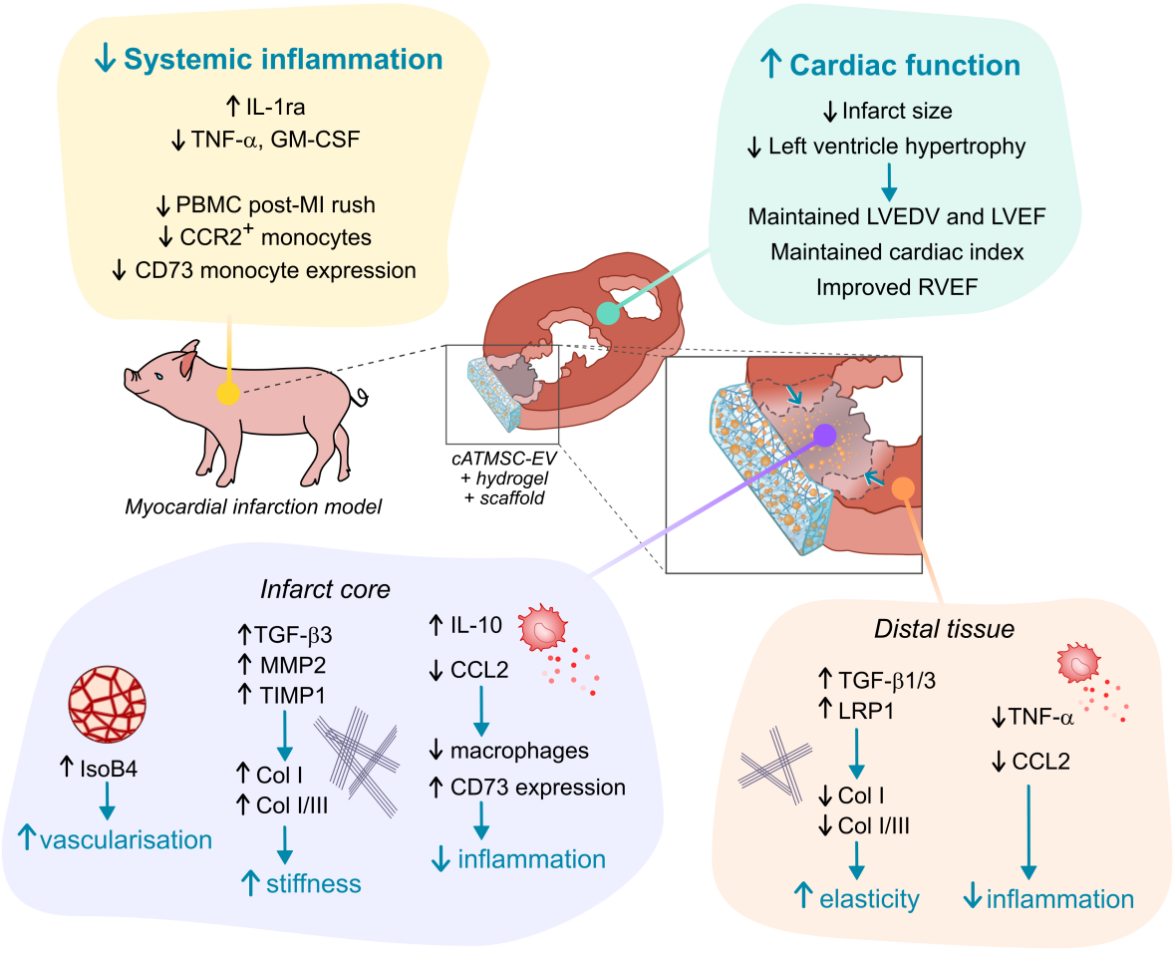
Schematic summary of results observed in cATMSC-EV Treated animals.

## Abbreviations

cATMSC: cardiac adipose tissue-derived MSC
cATMSC-EV: porcine cardiac adipose tissue MSC-EV
CCL2/MCP-1: chemokine (C-C motif) ligand 2 or monocyte chemoattractant protein 1
CI: cardiac index
cMRI: Cardiac magnetic resonance imaging
Col I/III: Collagen I/III
cTnI: cardiac troponin I
DAPI: 4′,6-diamidino-2-phenylindole dihydrochloride
ECG: electrocardiogram
ECM: extracellular matrix
EV: extracellular vesicles
FBS: foetal bovine serum
FDR: false discovery rate
GAPDH: glyceraldehyde-3-phosphate dehydrogenase
GM-CSF: granulocyte-macrophage colony-stimulating factor
GO: gene ontology
GUSB: glucuronidase beta
IL: interleukin
iLV mass: indexed left ventricle mass
iLVEDV: indexed left ventricle end-diastolic volumes
iLVESV: indexed left ventricle end-systolic volumes
iRVEDV: indexed right ventricle end-diastolic volumes
iRVESV: indexed right ventricle end-systolic volumes
IsoB4: isolectin B4
LGE mass: late gadolinium enhanced mass
LRP1: LDL receptor related protein 1
LV: left ventricle
LVEF: left ventricle ejection fraction
MI: myocardial infarction
MMP: metallopeptidases
MSC: mesenchymal stromal cell
NIR: near infrared
NTA: nanoparticle tracking analysis
PGK1: phosphoglycerate kinase 1
RT: room temperature
RVEF: right ventricle ejection fraction
SD: standard deviation
SEC: size exclusion chromatography
TGF-β: transforming growth factor beta
TIMP1: metallopeptidase inhibitor 1
TNF: tumour necrosis factor
