# Supplementary material for: Acellular cardiac scaffolds enriched with MSC-derived extracellular vesicles limit ventricular remodelling and exert local and systemic immunomodulation in a myocardial infarction porcine model
Source: Theranostics. 2022 Jun 6;12(10):4656–70. doi: 10.7150/thno.72289 (PMC9254233; doi:10.7150/thno.72289)
Supplement: Supplementary file 1 — Supplementary figures, tables, and methods. [file thnov12p4656s1.pdf]

Acellular cardiac scaffolds enriched with MSC-derived extracellular vesicles limit ventricular remodelling and exert local and systemic immunomodulation in a myocardial infarction porcine model

Marta Monguió-Tortajada, Cristina Prat-Vidal, Daina Martínez-Falguera, Albert Teis, Carolina Soler-Botija, Yvan Courageux, Micaela Munizaga-Larroudé, Miriam Moron-Font, Antoni Bayes-Genis, Francesc E. Borràs, Santiago Roura†, Carolina Gálvez-Montón†

Supplementary Material

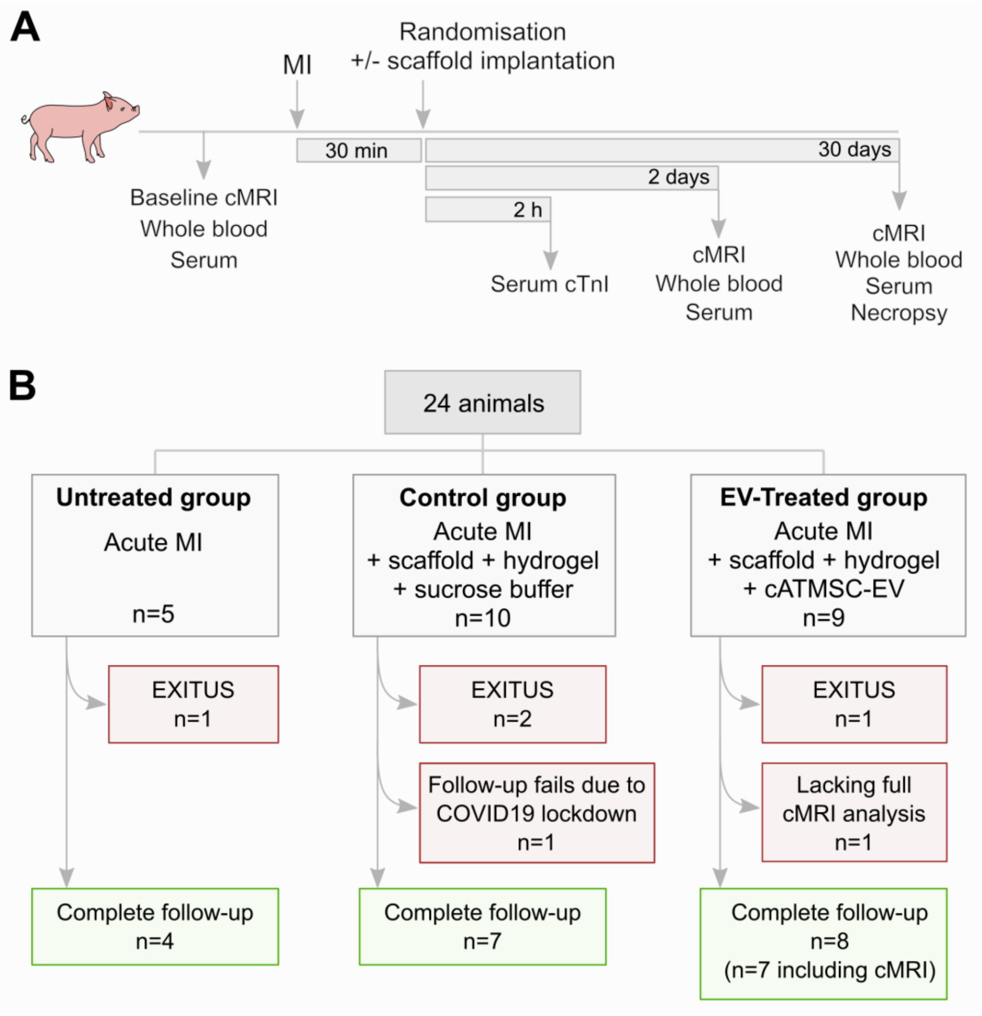

Figure S1. Schematic summary of (**A**) the animal experimentation journey and the (**B**) study groups and distribution of the 24 animals used in this study. MI: myocardial infarction; cATMSC-EV: cardiac adipose tissue mesenchymal stromal cells-derived extracellular vesicles; cTnI: cardiac troponin I; cMRI: cardiac magnetic resonance imaging.

Table S1. Antibodies used for peripheral immune cell study and tissue immunohistofluorescence analysis.

Antibody reactivity	Fluorochrome	Clone	Host	Reference	Manufacturer	Use
CD14	FITC	MIL2	Mouse IgG2b	MCA1218F	BioRad	FC
CD16	PE	G7	Mouse IgG1	MCA1971PE	BioRad	FC
CCR2	PE-Vio770	REA624	Human IgG1	130-103-901	Miltenyi Biotech	FC
CD163	NA	EDHu-1	Mouse IgG1	NB110-40686	Novus Biologicals	FC, IHC
Mouse	Cy3	Polyclonal	Goat	711-175-152	Jackson ImmunoResearch	
CD73	NA	Polyclonal	Rabbit IgG	NBP1-85740	Novus Biologicals	FC, IHC
Rabbit	Cy5	Polyclonal	Donkey	115-167-003	Jackson ImmunoResearch	FC
Rabbit	AF488	Polyclonal	Donkey	711-545-152	Jackson ImmunoResearch	IHC
CD3	NA	CD3-12	Mouse	MCA1477	BioRad	IHF
Mouse	AF488	Polyclonal	Donkey	712-485-153	Jackson ImmunoResearch	
CD25	NA	K231.3B2	Rat	MCA1736	BioRad	IHF
Rat	Cy3	Polyclonal	Goat	115-167-003	Jackson ImmunoResearch	
Isolectin-B4-biotin	NA	Polyclonal	NA	B-1205	Vector Labs	IHF
Streptavidin	AF488	Polyclonal	NA	S11223	Invitrogen	
Elastin	NA	Polyclonal	Rabbit	Ab21610	Abcam	IHF
Rabbit	Cy3	Polyclonal	Donkey	711-165-152	Jackson ImmunoResearch	
cTnI	NA	Polyclonal	Goat	Ab188877	Abcam	IHF
Goat	AF647	Polyclonal	Donkey	705-605-003	Jackson ImmunoResearch	

AF: Alexa Fluor; Cy: Cyanine; FITC: Fluorescein isothiocyanate; NA: not aplicable; PE: phycoerythrin; cTnI: cardiac troponin I; FC: flow cytometry; IHF: immunohistofluorescence.

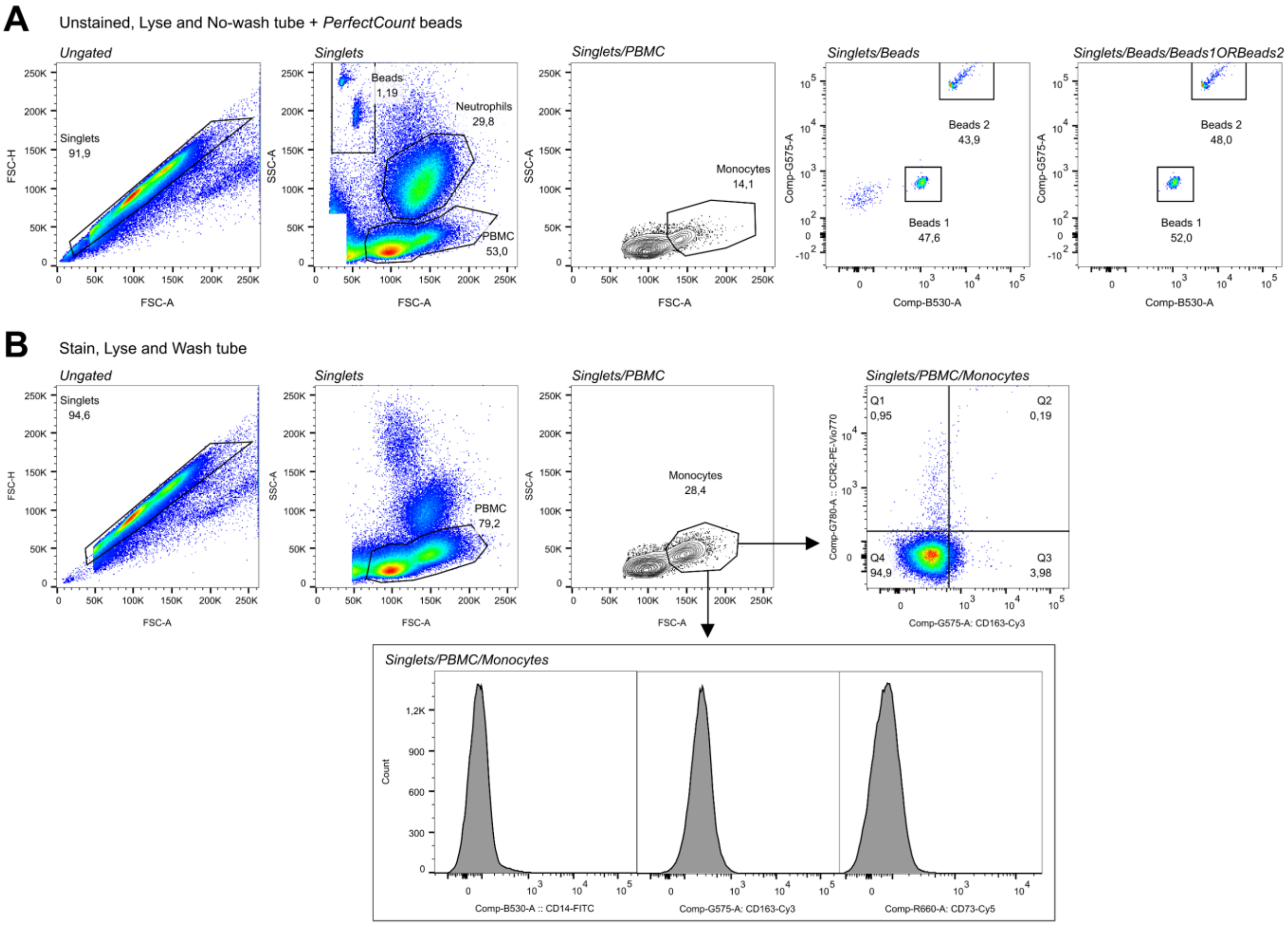

Figure S2. Gating strategy used for (**A**) counting the absolute number of peripheral blood cells and (**B**) phenotyping circulating monocytes. (**A**) Singlets were gated by FSC-A/FSC-H, then the neutrophil and PBMC populations were identified by size and complexity, from which monocytes were gated. Lymphocytes were calculated by subtracting the monocyte number from PBMC's. The absolute number of *PerfectCount* beads was used to calculate the absolute number of cells of each subset. They were gated as a Boolean "OR" gate of the two beads populations defined by their FITC (B530-A channel) and PE (G575-A channel) signal, and a 50:50 ratio (45:55 maximum difference) was ensured in every assay, according to manufacturer's instructions. (**B**) Monocytes were gated according to their distinctive FSC-A/SSC-A appearance from the *Singlets/PBMC* population. Then, the different % of cells expressing each marker and the median fluorescence intensity (MFI) in each channel were calculated.

Table S2. TaqMan® probes used in qPCR analysis.

Gene transcript	TaqMan® Probe[1]	Amplicon Length	Exon boundary
IL-10	Ss03382372_u1	87	3-3
TNF-α	Ss03391318_g1	73	3-4
CCL2/MCP-1	Ss03394377_m1	58	1-2
TGF-β1	Ss04955543_m1	57	5-6
TGF-β3	Ss03394351_m1	104	-
LRP1	Ss06917026_m1	57	4-5
MMP2	Ss03394318_m1	77	4-5
MMP9	Ss03392100_m1	58	12-13
TIMP1	Ss03381944_u1	105	5-5
GAPDH	Ss03375435_u1	75	4-4
PGK1	Ss03389144_m1	66	4-5
GUSB	Ss03387751_u1	62	12-12

[1]ThermoFisher Scientific reference.

IL-10: interleukin 10; TNF-α: tumor necrosis factor alpha; CCL2/MCP-1: chemokine (C-C motif) ligand 2 or monocyte chemoattractant protein 1; TGF-β1/3: transforming growth factor beta 1/3; LRP1: LDL receptor related protein 1; MMP2/9: matrix metalloproteinase 2/9; TIMP1: metallopeptidase inhibitor 1; GAPDH: glyceraldehyde-3-phosphate dehydrogenase; PGK1: phosphoglycerate kinase 1; GUSB: glucuronidase beta.

Table S3. Serum cTnI levels (pg/mL) in animals at baseline and 2 h after MI induction. Statistical differences according to paired two-way ANOVA (*p*=0.153) with Šídák's multiple comparisons test.

Group	Baseline		2 h post-MI		*p (time factor)*
	mean	SD	mean	SD	
Untreated	8.3	3.7	425.4	331.3	*0.0004*
Control	23.1	13.5	189.7	116.3	*0.0474*
EV-Treated	16.8	9.8	262.5	186.2	*0.0043*
p (group factor)	*0.085*		*0.118*		

Table S4. Cardiac parameters studied in cMRI analysis. Statistical differences are indicated between Baseline and 30 days post-MI data according to paired two-way ANOVA with Tukey's multiple comparisons test; **p*<0.05. The change observed at 30 days post-MI calculated as % from Baseline is indicated, with one arrow if the change is from 5 to a 10% difference, and two arrows for changes over the 10%. iLVEDV: indexed left ventricle end-diastolic volume; iLVESV: indexed left ventricle end-sistolic volume; LVEF: left ventricle ejection fraction; iRVEDV: indexed right ventricle end-diastolic volume; iRVEDV: indexed right ventricle end-sistolic volume; RVEF: right ventricle ejection fraction; CI: cardiac index; iLV mass: indexed left ventricle mass.

**iLVEDV** (%)									
**Group**	**Baseline**		**2 days post-MI**		**30 days post-MI**			**% 30 days post-MI to Baseline**	
	mean	SD	mean	SD	mean	SD	*p*	mean	SD
Untreated	103.0	7.4	109,4	10,7	110,7	8,4	0,532	↑ 8,3%	14,9%
Control	91.6	10.4	94,5	17,3	92,2	6,2	0,995	= 1,9%	14,8%
EV-Treated	103.9	14.6	106,2	21,2	99,7	17,8	0,717	= -3,9%	12,4%

**iLVESV** (mL)									
**Group**	**Baseline**		**2 days post-MI**		**30 days post-MI**			**% 30 days post-MI to Baseline**	
	mean	SD	mean	SD	mean	SD	*p*	mean	SD
Untreated	59,6	5,6	70,7	8,5	75,1	10,3	0,014 *	↑↑ 27,7%	28,3%
Control	54,3	5,0	58,4	12,1	58,0	6,9	0,611	↑ 7,5%	15,0%
EV-Treated	63,6	13,2	64,7	17,1	63,5	16,9	1,00	= 0,5%	18,8%

**LVEF** (mL)									
**Group**	**Baseline**		**2 days post-MI**		**30 days post-MI**			**% 30 days post-MI to Baseline**	
	mean	SD	mean	SD	mean	SD	*p*	mean	SD
Untreated	41.9	7.1	35,5	2,4	32,0	9,7	0,496	↓↓ -19,0%	39,4%
Control	40.5	4.5	38,2	6,6	37,0	7,6	0,688	↓ -6,6%	26,8%
EV-Treated	39,1	7,4	39,5	7,4	36,9	7,3	0,768	= -4,2%	19,3%

**iRVEDV** (%)									
**Group**	**Baseline**		**2 days post-MI**		**30 days post-MI**			**% 30 days post-MI to Baseline**	
	mean	SD	mean	SD	mean	SD	*p*	mean	SD
Untreated	83.2	12.7	94,2	9,6	95,4	13,3	0,407	↑↑ 18,5%	35,0%
Control	83.2	11.6	78,0	16,4	82,3	12,6	0,991	= 0,3%	19,4%
EV-Treated	79.7	12.1	88,4	19,1	80,9	9,2	0,987	= 3,6%	19,0%

**iRVESV** (mL)									
**Group**	**Baseline**		**2 days post-MI**		**30 days post-MI**			**% 30 days post-MI to Baseline**	
	mean	SD	mean	SD	mean	SD	*p*	mean	SD
Untreated	44,4	4,6	52,4	6,7	56,4	11,4	0,153	↑↑ 29,7%	35,4%
Control	42,6	8,2	41,8	12,6	45,4	8,8	0,824	↑ 8,7%	22,5%
EV-Treated	44,6	7,7	51,7	15,0	37,8	4,9	0,337	↓↓ -14,2%	11,3%

**RVEF** (mL)									
**Group**	**Baseline**		**2 days post-MI**		**30 days post-MI**			**% 30 days post-MI to Baseline**	
	mean	SD	mean	SD	mean	SD	*p*	mean	SD
Untreated	46.2	6.2	44,4	4,2	40,6	11,8	0,772	↓ -9,4%	34,2%
Control	48.9	6.6	47,0	8,0	44,9	5,9	0,461	↓ -6,7%	19,0%
EV-Treated	43.9	5.6	41,5	10,0	52,9	7,0	0,012 *	↑↑ 20,8%	12,5%

**CI** (mL)									
**Group**	**Baseline**		**2 days post-MI**		**30 days post-MI**			**% 30 days post-MI to Baseline**	
	mean	SD	mean	SD	mean	SD	*p*	mean	SD
Untreated	3.9	0.6	4,0	0,5	3,3	0,7	0,685	↓↓ -13,8%	27,8%
Control	4.3	1.1	3,6	0,5	3,8	0,7	0,698	= -3,9%	36,8%
EV-Treated	3.9	0.5	3,9	0,5	3,8	1,1	0,946	= 2,1%	30,3%

**iLV mass** (g)									
**Group**	**Baseline**		**2 days post-MI**		**30 days post-MI**			**% 30 days post-MI to Baseline**	
	mean	SD	mean	SD	mean	SD	*p*	mean	SD
Untreated	71.5	8.2	77,3	8,8	89,2	8,8	0,149	↑↑ 26,2%	21,3%
Control	75.6	7.7	80,6	13,1	84,8	13,2	0,070	↑↑ 11,9%	11,5%
EV-Treated	74.9	7.6	83,4	10,8	79,7	11,3	0,560	↑ 7,0%	16,5%

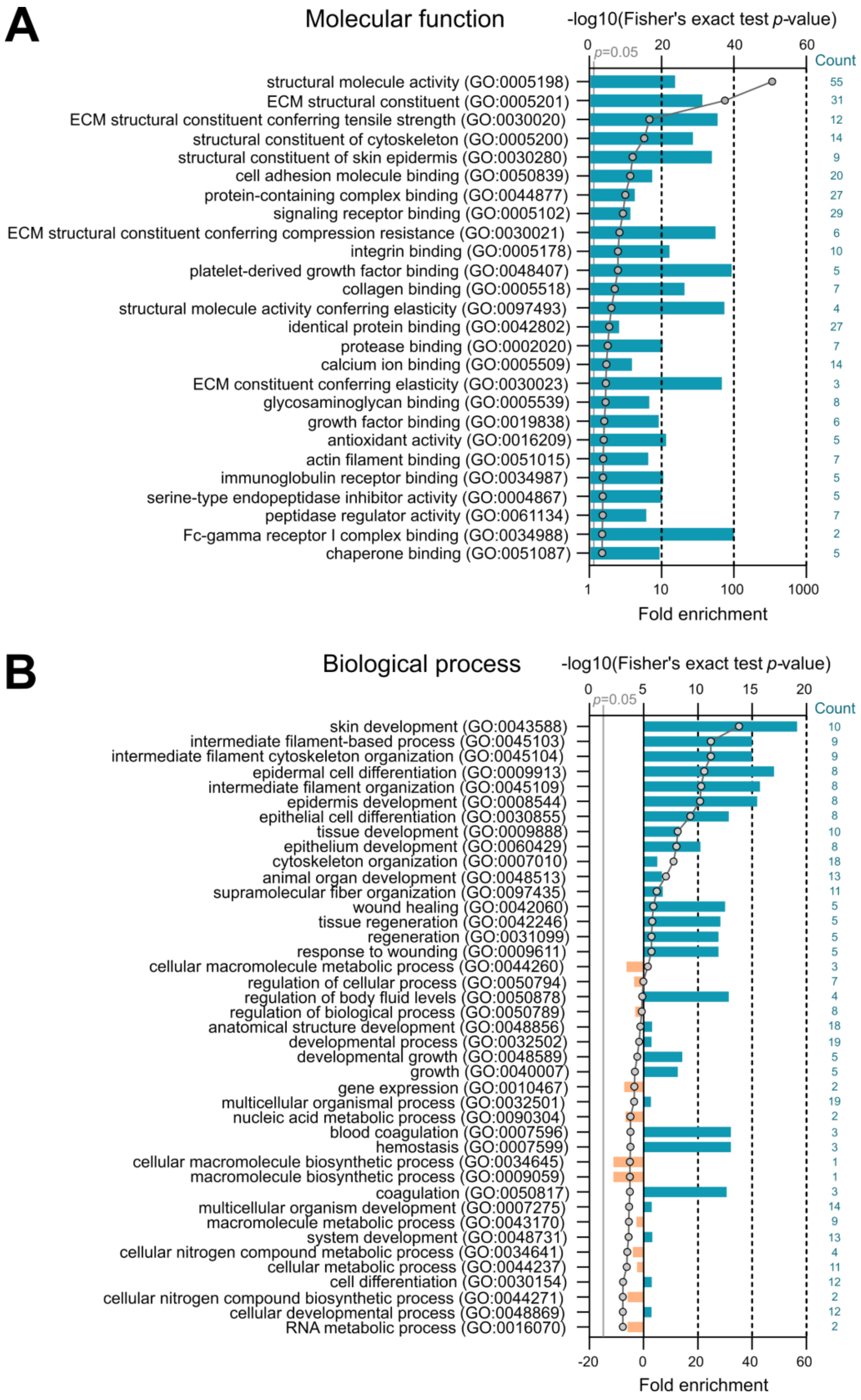

Figure S3. Functional enrichment analysis of decellularised scaffolds in terms of (**A**) molecular functions and (**B**) biological processes based on gene ontology (GO) terms. The GO terms with differential presence (p<0.05) in the proteomic analysis of decellularised pericardial scaffolds according to Fisher's exact test with false discovery rate (FDR) correction are depicted in the graphs.

Extended methods

EV delivery within cardiac scaffolds

Either mixture was laid over 2 cm^2 lyophilised, decellularised scaffolds, cut with a scalpel, to both rehydrate and fill the scaffolds with EV. After 15 min of seeding and scaffold rehydration at RT, 100 µL DMEM medium without phenol red (Gibco) was added over scaffolds to promote the salt-triggered peptide folding and consequent gel formation for 10 min. Then, scaffolds were washed twice with 2 mL Plasmalyte® 148 (Viaflo, Baxter) with 15 min incubation in between for pH balancing, and left at RT from 15 min to up to 2 h before *in vivo* implantation. Half of the produced EV-cardiac scaffolds were seeded with NIR815-labelled cATMSC-EV. To check the accuracy of the scaffold loading, they were scanned in the 800-nm channel in a Pearl Impulse Imager (Li-COR Biosciences - GmbH) just before implantation.

Non-invasive cardiac magnetic resonance imaging

All images were performed in a 3T state-of-the-art imaging system (Vantage Galan 3T, Canon Medical Systems) with all animals in prone position using a 16-element phased array coil (Atlas SPEEDER Body Coil) placed over the chest. Images were acquired during breath-holds with electrocardiographic gating. We used a segmented k-space steady state precession (SSFP) cine sequence (typically TR/TE 3.0/1.5 ms; 65º flip angle; 291x265 mm field of view (FOV); 352×320-pixel matrix, 8 mm slice thickness, 1302 Hz/pixel bandwidth) at 2, 3 and 4 chamber views and short axis from base to apex with no gap. Phase-contrast sequences (typically TR/TE 5.4/2.6 ms; 10º flip angle; 350x350 mm FOV; 8 mm slice thickness; 130-200 cm/sec VENC) were performed at the sino-tubular junction to calculate the aortic forward flow. Delayed enhancement images were acquired 10 to 20 min after IV injection of gadolinium-based contrast (Gd-DTPA; 0.2 mmol/kg) using a phase sensitive inversion recovery sequence (TR/TE 8.9/3.4 ms; 20º flip angle; 180-250 ms inversion time; 340×340mm FOV; 572x448-pixels matrix; 8 mm slice thickness, 140 Hz/pixel bandwidth) matching cine images positions. Inversion time was optimized to null the normal myocardium.

All images were reviewed and analysed off-line using a cMRI dedicated analysis software (Medis) by a level 3 cMRI expert blinded to the clinical data. Left and right ventricular (LV and RV, respectively) endocardial borders (papillary muscles were excluded) were manually traced in all short-axis cine images at the end-diastolic and end-systolic frames to determine end-diastolic and end-systolic volumes (EDV and ESV), respectively, using QMass (Medis). LV mass was calculated by subtracting the endocardial volume from the epicardial volume at end diastole and then multiplying by tissue density (1.05 g/mL). Left and right ejection fraction (LVEF and RVEF, respectively) were calculated. Calculation of forward aortic volume was performed using QFlow (Medis) tracing ROI at the aorta in phase-contrast sequences.

Background noise correction was performed at all images. The endocardial and epicardial contours on delayed enhancement images were also outlined manually. ROIs were then manually traced in the hyperenhanced area at place of maximum signal intensity and in the normal-appearing remote myocardium. As previously described, the areas of hyperenhanced myocardium were then automatically segmented by using a full-width at half-maximum (FWHM) algorithm with QMass. Two corrections were required for all automated ROIs. First, microvascular obstruction (defined as hypointensity within a hyperintense region in subjects with infarctions) was adjusted to be included as late gadolinium enhancement (LGE) if present. Second, any obvious blood pool or pericardial partial voluming and artefacts were further removed. Scar volume for each slice was calculated as: scar area × slice thickness. The scar mass was expressed as total scar volume ×1.05 g. Scar size was also expressed as a percentage of the total myocardial volume: (scar volume / myocardium volume) × 100.

Height, weight and heart rate of pigs was recorded at every cMRI scan. Body surface area (BSA) was calculated as previously described (Kelley et al. 1973). Cardiac index was calculated as (forward aortic volume × heart rate)/ BSA.
